# Synthesis, Docking and Biological Activities of Novel Hybrids Celecoxib and Anthraquinone Analogs as Potent Cytotoxic Agents

**DOI:** 10.3390/ijms151222580

**Published:** 2014-12-05

**Authors:** Maha S. Almutairi, Gehan H. Hegazy, Mogedda E. Haiba, Hamed I. Ali, Nagy M. Khalifa, Abd El-mohsen M. Soliman

**Affiliations:** 1 Pharmaceutical Chemistry Department, Faculty of Pharmacy, King Saud University, Riyadh 11451, Saudi Arabia; E-Mails: malmutairi30@gmail.com (M.S.A.); mogedda.haiba@yahoo.com (M.E.H.); 2 Pharmaceutical Chemistry Department, Faculty of Pharmacy, Cairo University, Cairo 11562, Egypt; E-Mail: gehan_hegazy@gmail.com; 3 Department of Pharmaceutical Sciences, Irma Lerma Rangel College of Pharmacy, Texas A&M Health Science Center, Kingsville, TX 78363, USA; E-Mail: hamed_ali37@yahoo.com; 4 Pharmaceutical Chemistry Department, Faculty of Pharmacy, Helwan University, Cairo 11790, Egypt; 5 Pharmaceutical Chemistry Department, Drug Exploration & Development Chair (DEDC), College of Pharmacy, King Saud University, Riyadh 11451, Saudi Arabia; 6 Department of Therapeutical Chemistry, Pharmaceutical and Drug Industries Division, National Research Center, Dokki, Cairo 12622, Egypt; E-Mail: solimanmohsen@yahoo.com

**Keywords:** antitumor, anthraquinone, celecoxib, HEPG2, docking, protein kinase activities

## Abstract

Herein, novel hybrid compounds of celecoxib and 2-aminoanthraquinone derivatives have been synthesized using condensation reactions of celecoxib with 2-aminoanthraquinone derivatives or 2-aminoanthraquinon with celecoxib derivatives. Celecoxib was reacted with different acid chlorides, 2-chloroethylisocyanate and bis (2-chloroethyl) amine hydrochloride. These intermediates were then reacted with 2-aminoanthraquinone. Also the same different acid chlorides and 2-chloroethylisocyanate were reacted with 2-aminoanthraquinone and the resulting intermediates were reacted with celecoxib to give isomers for the previous compounds. The antitumor activities against hepatic carcinoma tumor cell line (HEPG2) have been investigated *in vitro*, and all these compounds showed promising activities, especially compound **3c**, **7**, and **12**. Flexible docking studies involving AutoDock 4.2 was investigated to identify the potential binding affinities and the mode of interaction of the hybrid compounds into two protein tyrosine kinases namely, SRC (Pp60v-src) and platelet-derived growth factor receptor, PDGFR (c-Kit). The compounds in this study have a preferential affinity for the c-Kit PDGFR PTK over the non-receptor tyrosine kinase SRC (Pp60v-src).

## 1. Introduction

Cancer is one of the most widespread serious diseases. It is characterized by uncontrolled growth of abnormal cells. The growth and metastasis of cancer cells are dependent on angiogenesis; Therefore, affecting angiogenesis will be of great importance in inhibition of tumor growth, invasion, and metastasis [1]. Prostaglandins are inflammatory mediators which are highly expressed in cancer angiogenesis. Prostaglandins are derived from arachidonic acid by either Cox-1 or Cox-2 [2,3,4]. Cox-2 modulates cell proliferation and the apoptosis process, which plays an important role in cancer progression both in human and animal models malignancies [5]. It was shown that Cox-2 is over expressed in various cancers and this over expression protects cancer cells against several apoptotic stimuli [6]. A further study showed that knock-out of the Cox-2 gene could inhibit tumorgenesis [7]. Several new clinical studies were performed on celecoxib, a selective Cox-2 inhibitor which proved its effectiveness in many types of cancer including colorectal cancer, prostate cancer, breast cancer, non-small cell lung cancer [8,9], ovarian cancer [10], head and neck squamous cell carcinoma [11]. Another potential mechanism for anticancer agents is by targeting the DNA by intercalating agents such as anthraquinones [12]. These compounds have long been used as effective anticancer drugs against a broad spectrum of tumors. Depending on their chemical structures, anthraquinone drugs can kill tumor cells by diverse mechanisms including apoptosis. Apoptosis has been suggested to play an important role in the therapeutic activity of anthraquinone drugs on tumor cells. The apoptotic process triggered by mitoxantrone, an anthraquinone derivative, was mediated by caspase-3 activation [13]. Moreover, anthraquinone derivatives can inhibit tumor associated angiogenesis through inhibition of extracellular signal-regulated kinase [14]. Protein-tyrosine kinases (PTKs) are the key intermediates in cell signaling pathways that regulate cell growth and apoptosis. Altered functions of individual protein kinases result in numerous pathological conditions, including uncontrolled cell proliferation [15]. Tyrosine kinase inhibitors can be considered as a target for anti-angiogenesis and applied as a new model of cancer therapy [16]. With the emergence of *in vitro* biochemical assays and the development of biochemical and genetic studies, the drug discovery paradigm has shifted from the animal model screening approach to the target-focused ligand discovery model. This concept has led to many drugs reaching the market [17]. Computer prediction of the interaction between enzymes and small molecules has now advanced to the point that it allows accurate prediction of bound conformations and binding constants. Docking of the molecules into their respective 3D macromolecular targets is a widely used method for lead optimization. One of the most well-known docking programs is AutoDock [18]. In recent years focusing on new anticancer drugs has become a major challenge. The combination of two different mechanisms in one compound is one of the major tools to find out more potentially active anticancer drugs. Thus, in the current study, we combine the two moieties celecoxib and anthraquinones in one hybrid structure aiming to increase their angiogenic effect to produce new potent anticancer agents.

## 2. Results and Discussion

### 2.1. Chemistry

**Scheme 1:**
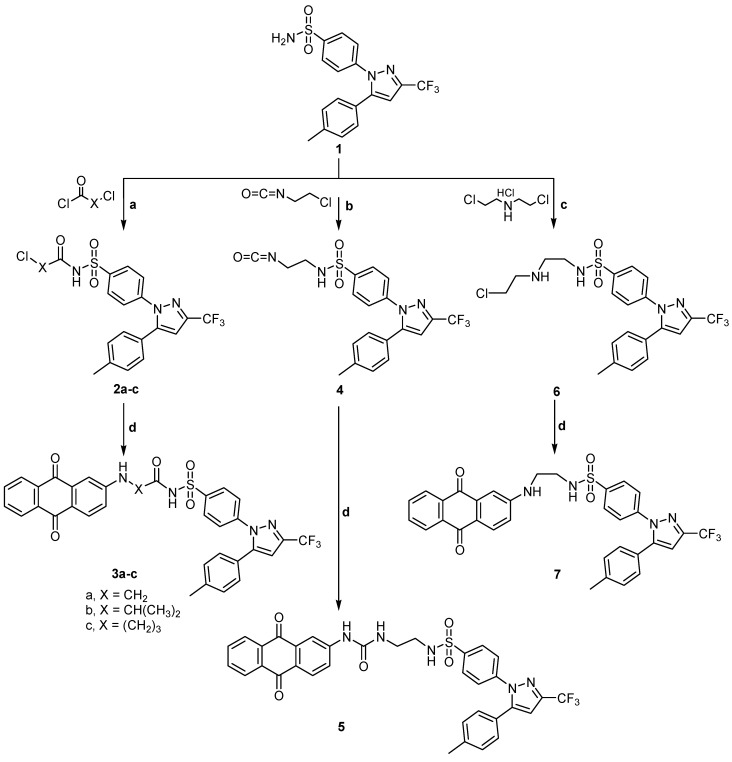
General methods for the preparation of **2a**–**c**, **3a**–**c**, **4**, **5**, **6** and **7**, Reagents and conditions: (**a**) Acid chloride, reflux, 1 h; (**b**) 2-Chloroethylisocyanate, reflux, 1 h; (**c**) 2,2'-Dichloroethylamine hydrochloride, dry toluene, reflux, 4 h; and (**d**) 2-Aminoanthraquinone, Dimethyl formamide (DMF), reflux, 4–8 h.

Compounds **2a** was obtained according to the reported method [19] while compounds **2b**, **c** were obtained by fusion of celecoxib **1** and 2-chloropropanoyl chloride, or chlorobutyryl chloride. Compounds **2a**–**c** were subjected to condensation with 2-aminoanthraquinone **8** to give 2- or 4-((9,10-dihydro-9,10-dioxoanthracen-2-yl)amino)-*N*-(4-(5-(*p*-tolyl)-3-(trifluoromethyl)-1*H*-pyrazol-1-yl)phenyl)sulfonyl) alkanamides **3a**–**c**. Also, compound **4** was prepared by the same method on reaction of celecoxib **1** and 2-chloroethylisocyanate. The key intermediate **4** was then reacted with 2-aminoanthraquinone **8** to give the desired product **5**. Moreover the intermediate **6** was prepared by reaction of celecoxib **1** with 2,2'-dichlorodiethylamine hydrochloride in dry toluene. It was then reacted with 2-aminoanthraquinone **8** to produce **7** (Scheme 1).

**Scheme 2:**
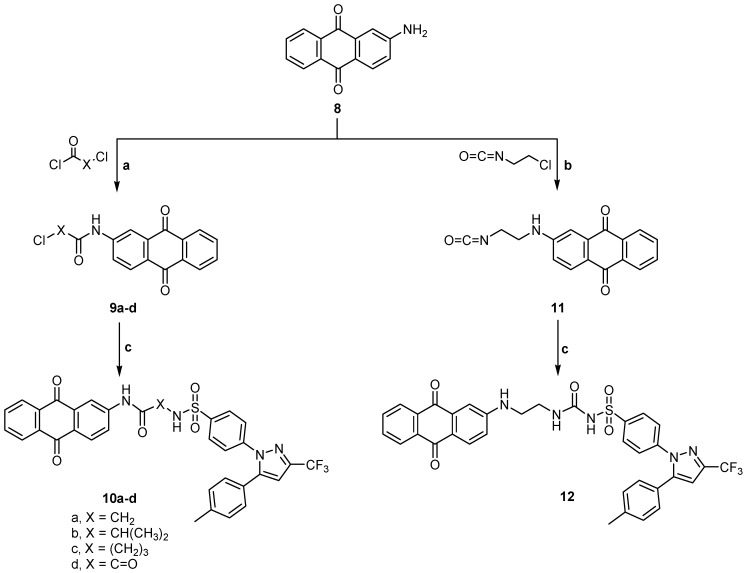
General methods for the preparation of **9a**–**d**, **10a**–**d**, **11** and **12**. Reagents and conditions: (**a**) Acid chloride, reflux, 1 h; (**b**) 2-Chloroethylisocyanate, reflux, 1 h; and (**c**) Celecoxib, ethanol, DMF, reflux, 4–5 h.

On the other hand 2-aminoanthraquinone **8** was reacted with the same series of chloroacyl chlorides or oxalyl dichloride to give the intermediates 2- or 4-chloro-*N*-(9,10-dihydro-9,10-dioxoanthracen-2-yl) alkanamides **9a**–**c**, **9a** was obtained according to a previously reported method [20] or 2-((9,10-dihydro-9,10-dioxoanthracen-2-yl)amino)-2-oxoacetyl chloride **9d** according to the reported method [21], which on reaction with celecoxib **1** gave the target compounds **10a**–**d**. Compound **8** was also reacted with 2-chloroethylisocyanate to give the intermediate **11** according to the reported method [12] which was then reacted with celecoxib **1** to give the final compound **12** (Scheme 2). It is clear that compounds **3a**–**c** are isomers for compounds **10a**–**c**, while compound **10d** can be obtained by applying the same method applied for preparation of compounds **3a**–**c**. Again compound **5** is an isomer for compound **12**. This may give an idea about the effect of isomerism on the activity of the final compounds.

### 2.2. Pharmacological Screening

It is well known that chemotherapy aims to destroy the cancer cells with various types of chemicals. The substances used are supposed to target mainly the cancer cells and doses are calculated to minimize the collateral damage to surrounding tissues, which nevertheless occurs [22]. This kind of treatment increases the entropy of the organism, suppresses the immune system, and forms a toxic cell environment which may destroy surrounding healthy cells [23], so it is important to minimize curing doses to the least amount possible to minimize the side effects of these drugs. The antitumor activities of the new compounds **3a**–**c**, **7**, **10a**–**d** and **12** were assessed against the HEPG2 cancer cell line in comparison to the traditional anticancer drugs 5-flurouracil (5-FU) and doxorubicin (DOX). Regarding the antitumor activity results, all of the selected compounds showed reasonable antitumor activity in comparison to 5-FU and DOX in concentration ranging from 3.92–9.38 µg/mL. Table 1 shows the cytotoxic activity of the selected newly synthesized derivatives, where compounds **3c**, **7** and **12** were the most active derivatives with IC_50_ equals 3.74, 4.31 and 3.92 respectively.

Moreover, the biochemical effects of the selected derivatives on some enzymes such as alanine and aspartate aminotransferases (ALT and AST) and alkaline phosphatase (ALP), in addition to total lipids, cholesterol, triglycerides, bilirubin, albumin, globulin and creatinine in serum of mice were investigated. The study of the induced biochemical parameters of most of the tested compounds in mice showed insignificant differences relative to the control group which indicates a moderate safety margin for the selected compounds as shown in Table 2, Table 3 and Table 4.

**Table 1:** The antiproliferative effects of anthraquinone derivatives against hepatic carcinoma tumor cell line (HEPG2).

Compound	IC_50_ (μg·mL^−1^)
**3a**	6.53
**3b**	9.38
**3c**	3.74
**7**	4.31
**10a**	8.48
**10b**	7.43
**10c**	8.54
**10d**	6.59
**12**	3.92
**5-Flurouracil**	5.00
**Doxorubicin**	3.56

**Table 2:** Biochemical effects (Mean ± SD (Standard Deviation) of treatment with 5-flurouracil (5-FU), doxorubicin (DOX), and the anthraquinone derivatives on serum ALT, AST, and ALP in mice.

Compounds	ALT (IU/mL)	AST (IU/mL)	ALP (k.k./dL)
**3a**	38.53 ± 6.50 ***	111.59 ± 12.80 **	19.99 ± 4.39 ***
**3b**	60.80 ± 9.20 *	157.28 ± 20.30 *	43.25 ± 7.08 *
**3c**	67.39 ± 11.00 *	146.40 ± 28.10 *	36.90 ± 9.80 *
**7**	39.10 ± 8.40 ***	126.20 ± 12.10 **	18.76 ± 6.43 ***
**10a**	45.51 ± 4.23 ***	108.66 ± 4.61 ***	21.77 ± 3.50 **
**10b**	54.2 ± 11.05 *	146.50 ± 28.90 *	46.47 ± 10.87 *
**10c**	78.10 ± 13.30 *	141.20 ± 32.04 *	31.46 ± 9.45 *
**10d**	48.07 ± 6.16 ***	112.09 ± 8.83 ***	17.79 ± 3.06 ***
**12**	51.88 ± 11.50 **	113.00 ± 9.52 **	21.09 ± 3.48 **
**Control**	43.50 ± 2.03	108.32 ± 4.19	17.70 ± 1.10
**5-Flurouracil**	51.47 ± 9.02 *	130.43 ± 8.92 *	25.49 ± 6.03 *
**Doxorubicin**	59.26 ± 12.03 *	147.23 ± 16.34 *	30.32 ± 5.14 *

* *p* < 0.001: Highly significant; ** *p* < 0.01: Significant; *** n.s.: Non significant; ALT: Alanine amino transferase; AST: Aspartate amino transferase; ALP: Alkaline phosphatase; and k.k./dL: Kind & King Unit/Dalton.

**Table 3:** Biochemical effects (Mean ± SD) of treatment with 5-FU, DOX, and anthraquinone derivatives on total lipids, cholesterol, triglycerides, and bilirubin in mice.

Compounds	Total Lipids (mg/dL)	Cholesterol (mg/dL)	Triglycerides (mg/dL)	Bilirubin (mg/dL)
**3a**	336.40 ± 19.10 ***	97.20 ± 9.90 ***	117.90 ± 18.40 **	0.67 ± 0.03 ***
**3b**	379.20 ± 37.80 *	127.50 ± 25.10 *	136.10 ± 27.09 *	0.97 ± 0.05 *
**3c**	321.70 ± 18.90 ***	92.80 ± 14.30 ***	115.40 ± 8.70 ***	0.63 ± 0.05 ***
**7**	329.60 ± 14.40 ***	97.40 ± 18.60 ***	114.90 ± 10.70 ***	0.64 ± 0.02 ***
**10a**	313.70 ± 31.20 ***	94.80 ± 18.60 ***	117.31 ± 21.60 ***	0.55 ± 0.06 **
**10b**	374.60 ± 36.80 *	111.40 ± 16.50 **	97.40 ± 9.60 ***	1.09 ± 0.60 *
**10c**	375.26 ± 27.80 *	111.80 ± 31.40 **	157.90 ± 35.70 *	0.74 ± 0.03 **
**10d**	328.50 ± 22.70 ***	96.39 ± 17.10 ***	115.51 ± 18.10 ***	0.54 ± 0.05 **
**12**	369.30 ± 26.30 *	109.90 ± 18.60 **	114.20 ± 18.40 **	0.73 ± 0.16 **
**Control**	323.41 ± 27.10	94.32 ± 13.50	108.70 ± 16.80	0.63 ± 0.04
**5-Flurouracil**	378.20 ± 31.40 *	105.90 ± 11.70 *	126.50 ± 19.40 *	0.75 ± 0.10 *
**Doxorubicin**	366.70 ± 6.10 *	109.30 ± 14.20 *	137.80 ± 17.10 *	0.81 ± 0.19 *

* *p* < 0.001: Highly significant; ** *p* < 0.01: Significant; and *** n.s.: Non significant.

**Table 4:** Biochemical effects of treatment with 5-FU, DOX, and anthraquinone derivatives on serum albumin, globulin and creatinine in mice.

Biochemical Parameters	Albumin (mg/dL)	Globulin (mg/dL)	A/G Ratio	Creatinine (mg/dL)
**3a**	5.96 ± 0.4 ***	4.3 ± 0.69 ***	1.46 ***	0.62 ± 0.08 ***
**3b**	6.43 ± 0.44 **	6.46 ± 0.8 **	1.001 *	0.73 ± 0.06 **
**3c**	5.65 ± 0.69 ***	4.67 ± 1.09 ***	1.13 ***	0.66 ± 0.07 ***
**7**	5.92 ± 0.86 ***	4.73 ± 0.87 ***	1.15 ***	0.85 ± 0.08 **
**10a**	5.95 ± 0.78 ***	5.16 ± 0.7 ***	1.15 ***	0.72 ± 0.08 ***
**10b**	6.81 ± 0.47 **	6.79 ± 0.7 **	1.02 *	1.62 ± 0.07 *
**10c**	10.26 ± 1.31 *	8.97 ± 0.9 *	1.14 **	0.73 ± 0.03 ***
**10d**	5.53 ± 0.71 ***	4.88 ± 1.01 ***	1.13 ***	0.68 ± 0.04 ***
**12**	7.4 ± 0.59 **	6.65 ± 0.81 **	1.006 *	0.8 ± 0.09 **
**Control**	5.63 ± 0.51	4.32 ± 0.9	1.3	0.69 ± 0.03
**5-FU**	6.49 ± 0.92 **	5.75 ± 0.8 **	1.13 **	0.81 ± 0.06 **
**DOX**	6.37 ± 0.85 **	5.91 ± 0.63 **	1.078 **	0.78 ± 0.04 **

* *p* < 0.001: Highly significant; ** *p* < 0.01: Significant; and *** n.s.: Non significant.

Data in Table 2 present the liver enzymatic activities (ALT, AST and ALP) in serum of control and treated groups of mice. The results showed that the values recorded for AST and ALT were significantly higher (*p* < 0.001) with 5-FU and DOX treated groups of mice than the control. On the other hand, treatment with the new compounds tested, caused inverse effects, where some values recorded for AST and ALT were non significant (n.s.) or slightly higher (*p* < 0.01) in comparison to control. Moreover, the recorded data showed that ALP activities were significantly increased (*p* < 0.001) with the treatment with 5-FU and DOX, while there were no significant changes in ALP activities upon treatment with some of the compounds.

Data listed in Table 3 demonstrate the comparison between the levels of total lipids, cholesterol, triglycerides and bilirubin in serum of treated mice and the control group. It can be deduced from the present data that 5-FU and DOX caused a significant increase in the level of these parameters while treatment with the new derivatives tested showed moderate or no significant changes.

Table 4 represents a comparison between the levels of albumin, globulins and creatinine in serum of control and treated groups of mice. It is clear from these results that there was a slight increase in the level of albumin and creatinine and globulins in the 5-FU and DOX treated groups of mice while there were moderate or non significant changes in the other treated groups.

Based on these findings in the present work, the compounds in this study would have better biological activity as anti-proliferative agents with less toxic side effects.

### 2.3. Molecular Docking Study

Molecular docking is a frequently used tool in computer-aided structure-based rational drug design. It evaluates how small molecules called (e.g., drug candidates) and the target macromolecule (receptor or enzyme) fit together. AutoDock Tools (ADT) is a program package of automated docking tools that is available from http://autodock.scripps.edu/. It is designed to predict how small molecules bind to a target protein of known 3D-structure. Besides generating binding energies in these docking studies, the position of the ligand in the host’s binding site can be visualized. It can be useful for developing better drug candidates and also for understanding the nature of the binding. In this study, two classes of compounds were involved, including: first, the non-receptor tyrosine kinase SRC (Pp60v-src; pdb code: 1skj). Second, the c-Kit receptor PTK: Platelet-derived growth factor receptor (PDGFR, c-Kit; pdb code: 1t46). The deregulated c-Kit kinase activity is implicated in the pathogenesis of human cancers. The activity of the c-Kit receptor protein-tyrosine kinase is tightly regulated in normal cells [24].

#### 2.3.1. Evaluation of Docking Performance and Accuracy into Different PTKs

**Figure 1:**
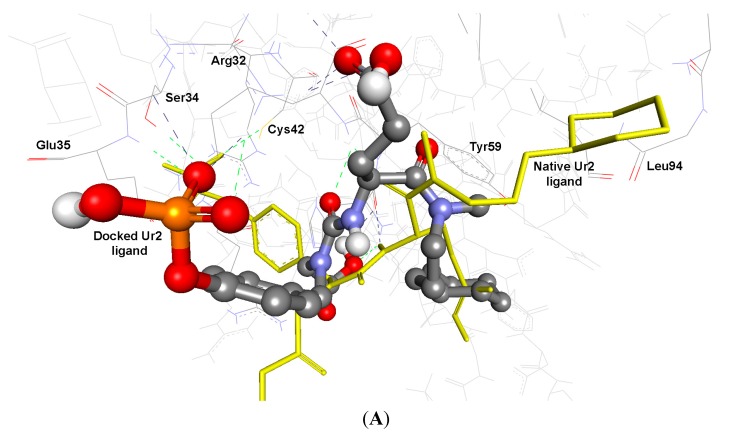

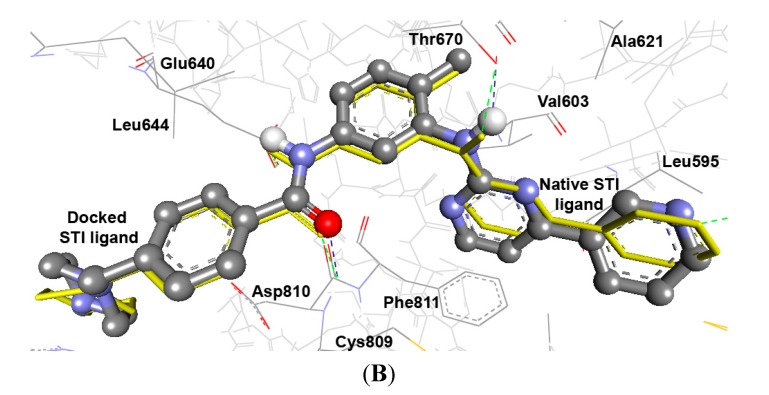
Validation of AutoDock 4.2 programs by docking of the native co-crystallized ligands UR2 and STI of 1skj and 1t46 into their binding sites, in (**A**) and (**B**), respectively. The native ligands are shown in yellow sticks, while the docked ones are shown in balls and sticks, colored by element. Their hydrogen bonds are shown as blue and green dotted lines, respectively. The docked STI ligand seems exactly superimposed on the native one within 0.39 Å RMSD, whereas UR2 ligand seems partially overlapped on the native one within 1.84 Å RMSD.

As cited in (Table 5 and Table 6), the Root Mean Square Deviation (RMSD) values of the co-crystallized ligands UR2 and 1skj in the two PTKs involved, are 1.84 and 0.39 Å, respectively. It was found that the docked ligands are exactly superimposed with the originally embedded native ligands especially into c-Kit PTK, not only for the best-scored conformations, but also for all the docked conformations (Figure 1A,B). The docked STI-571 ligand (Imatinib or Gleevec), 4-(4-methylpiperazin-1-yl-methyl)-*N*-[4-methyl-3-(4-pyridin-3-ylpyrimidin-2-ylamino)phenyl]benzamide, into its c-Kit receptor PTK (pdb code: 1t46), exhibited the best RMSD of 0.39 Å, and a low binding free energy (∆*G*_b_) of −15.85 kcal/mol. The docking results of the natively embedded ligands of PTKs were reasonably well comparable and well correlated to their biological methods [25,26]; Hence these results indicated that flexible docking involving AutoDock 4.2 under our experimental parameters seems to be accurate, with small RMSD values highly resembling the biological co-crystallization.

#### 2.3.2. Docking Study of the Synthesized Compounds

Docking of our novel compounds into the nonreceptor tyrosine kinase SRC (Pp60v-src; pdb code: 1skj), indicated that compounds revealing the best results include compounds: **3a**, **10a**, **10b**, and **10d**. They exhibited the lowest binding free energies (∆*G*_b_) being: −11.57, −10.89, −11.17 and −11.73 kcal/mol, respectively with RMSD range of 1.43–4.20 Å. Moreover, they bind into PTK through up to five hydrogen bonds, involving their common moieties being: Anthraquinone C=O, SO_2_NH, and O=CNH which were bound to the commonly involved amino acids being: Arg12 (NH), His58 (C=O), Lys60 (NH), Thr72 (OH), and Arg62 (NH), as cited in Table 5.

**Table 5:** The flexible docking results (AutoDock 4.2), regarding the binding free energies (Δ*G*_b_) and inhibition constants (*K*_i_) of compounds docked into PTK (1skj).

Comp.	∆*G*_b_^a^ (kcal/mol)	*K_i_* ^b^ (nM)	Hydrogen Bonds between Atoms of Compounds and Amino Acids of PTK (1skj)				RMSD ^c^ (Å)
			Atoms of Compounds	Amino Acids	Distance (Å)	Angle (°)	
**3a**	−11.57	3.31	Ph–N	HN of Lys60	2.48	154.9	1.43
**3b**	−10.86	10.94	Anthraquinone C=O	HN of His58	2.27	140.6	4.19
			Terminal C–F	HN of Arg12	2.29	168.3	
**3c**	−10.56	18.04	–SO_2_NHC=O	HN of Lys60	1.90	172.1	5.60
**7**	−9.86	59.49	S=O ^1^	^1^HN of Arg12	2.39	134.4	5.72
			S=O ^2^	^1^HN of Arg12	2.24	134.9	
			S=O ^2^	^2^HN of Arg12	2.07	140.1	
**10a**	−10.89	10.40	Anthraquinone C=O	HN of His58	2.31	123.8	4.20
**10b**	−11.17	6.45	Anthraquinone C=O	HN of Arg12	1.91	135.8	2.01
			CONH	O=C of His58	1.91	142.0	
			SO2NH	O=C of His58	2.22	150.1	
			SO2HN	HN of Lys60	2.47	164.0	
			Pyrazole=N	HN of Arg62	2.47	100.8	
**10c**	−9.69	78.94	Anthraquinone C=O	HN of Arg62	2.07	159.3	5.80
			–S=O ^1^	HN of Lys60	1.92	144.1	
			–S=O ^2^	HN of Lys60	1.91	126.4	
			SO_2_NH	O=C of His58	2.24	150.2	
**10d**	−11.73	2.53	Pyrazole=N	HN of Lys60	1.97	155.1	1.67
**12**	−9.85	59.82	CONH	O=C of His58	1.74	142.4	3.87
			HNS=O	HN of Lys60	1.97	162.5	
**UR2 ^d^**	−11.61	3.11	Terminal COO	HN of His58	2.27	146.4	1.84
			HNC=O	HN of Lys60	2.32	124.5	
			Terminal P=O ^1^	HN of Arg62	2.44	122.4	
			Terminal P=O ^2^	HN of Arg62	2.35	140.5	

^a^ Binding free energy; ^b^ Inhibition constant; ^c^ Root mean square deviation; ^d^ 4-[3-Carboxymethyl-3-(4-phosphonooxybenzyl)ureido]-4-[(3-cyclohexylpropyl)methylcarbamoyl]butyric acid; ^1^ and ^2^ indicate the specific atom involved in the hydrogen binding when there is two or more similar groups.

**Figure 2:**
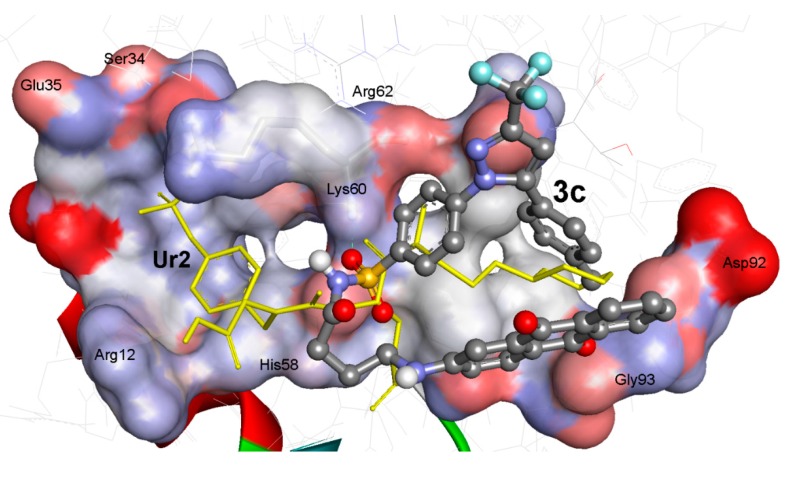
The binding affinities of compounds **3c**; Ball and stick, colored by elements, (grey = carbon, red = oxygen, blue = nitrogen, and yellow = sulfur) involving flexible docking into 1skj PTk. Compound **3c** (Δ*G*_b_: −10.56 kcal/mol; RMSD: 5.60 Å) is partially superimposed onto the Ur2 native ligand; Yellow sticks, and it exhibited one hydrogen bonds (green dashed lines) with Lys60. PTK binding site is shown with solid surface.

As illustrated in Figure 2, compound **3c** possess high potential binding affinity (∆*G*_b_: −10.56 kcal/mol) into the binding site of the 3D macromolecule (PTK, 1skj). Its high affinity is presumably attributed to formation of one hydrogen bonds between its sulfone S=O and HN of Lys60 amino acid of the binding site. Compound **3c** was docked partially superimposed onto the native ligand (UR2) into the binding pocket of PTK within RMSD being 5.60 Å.

The docking of our compounds into c-Kit receptor PTK: Platelet-derived growth factor receptor (PDGFR, c-Kit, pdb code: 1t46), indicated that compounds revealing the best results include compounds: **3a**, **10a**, **10b**, and **10d**. They exhibited the lowest binding free energies (∆*G*_b_) of −13.20, −13.37, −12.63, and −14.22 kcal/mol, respectively with RMSD range of 4.29–5.98 Å. They bind into PTK through up to three hydrogen bonds, involving their common moieties being: Ph–NH, Ph–NH–C=O, anthraquinone C=O, and SO_2_NH which bound to the commonly involved amino acids being: Glu640 (C=O), Thr670 (OH), Asp810 (C=O, NH), and Lys623 (NH) as cited in Table 6.

**Table 6:** The flexible docking results (AutoDock 4.2), regarding the binding free energies (Δ*G*_b_) and inhibition constants (*K*_i_) of compounds docked into c-Kit PTK (1t46).

Comp.	∆*G*_b_^a^ (kcal/mol)	*K_i_* ^b^	Hydrogen Bonds between Atoms of Compounds and Amino Acids of PTK (1t46)				RMSD ^c^ (Å)
			Atoms of Comp.	Amino Acids	Distance(Å)	Angle (°)	
**3a**	−13.20	212.15 pM	–SO_2_NH	O=C of Glu640	2.46	175.8	5.98
**3b**	−9.91	54.76 nM	Ph–NH	O=C of Asp810	1.92	153.6	10.0
**3c**	−10.56	18.09 nM	d				10.05
**7**	−9.83	62.78 nM	Ph–NH	O=C of Asp810	2.34	114.2	9.46
			Ph–NHCH_2_NH	O=C of Asp81	2.26	112.1	
			Ph–NHCH_2_NH	O–C of Asp810	1.96	145.7	
			Terminal C–F	HN of Arg791	2.12	122.6	
**10a**	−13.37	157.86 pM	Ph–NH	O=C of Asp810	2.19	122.4	4.36
			Ph–NH	O=C of Glu640	1.75	105.4	
**10b**	−12.63	555.42 pM	Anthraquinone C=O	HN of Asp810	2.35	129.6	5.66
			Ph–NHC=O	HN of Lys623	2.41	103.0	
**10c**	−12.01	1.57 nM	Anthraquinone C=O ^a^	HO of Thr670	2.39	100.6	5.31
			Anthraquinone C=O ^b^	O=C of Glu640	2.02	144.5	
			Ph–NH	HN of Lys623	2.38	124.9	
**10d**	−14.22	37.78 pM	Anthraquinone C=O	HO of Thr670	2.22	110.1	4.29
			Ph–NH	O=C of Glu640	1.91	162.8	
			Ph–NHC=O	HN of Asp810	2.33	104.5	
**12**	−11.86	2.02 nM	Ph–NH	CO of Asp792	2.05	130.2	10.84
			Terminal C–F	NH of Ile789	2.29		
**STI ^e^**	−15.85	2.41 pM	Ph–C=O	HN of Asp810	2.25	146.3	0.39
			Ph–NH	OH of Thr670	2.13	130.9	

^a^ Binding free energy; ^b^ Inhibition constant; ^c^ Root mean square deviation; ^d^ No hydrogen bond detected; ^e^ 4-(4-Methylpiperazin-1-yl methyl)-*n*-[4-methyl-3-(4-pyridin-3-yl pyrimidin-2-yl amino)phenyl]benzamide.

To investigate the potential PTK inhibition, the comparatively effective antiproliferative derivatives against HEPG2, namely **10d** and **12** were docked into PTK (1t46) as shown in detail in (Figure 4). Compound **10d** revealed the highest binding affinities into the binding sites of PTK (∆*G*_b_: −14.22 kcal/mol) with three hydrogen bonds between its anthraquinone C=O, Ph–NH, and Ph–NHC=O moieties and Glu640 (C=O), Thr670 (OH), and Asp810 (NH) amino acids. Additionally, the RMSD is of 4.29 Å and it is seen docked deeply into the binding pocket. Also, compound **12** was tightly bound into PTK through two hydrogen bonds via its Ph-NH and terminal C–F groups and Asp792 (C=O) and Ile789 (NH) with (∆*G*_b_: −11.86 kcal/mol) and RMSD of 10.84 Å.

**Figure 3:**
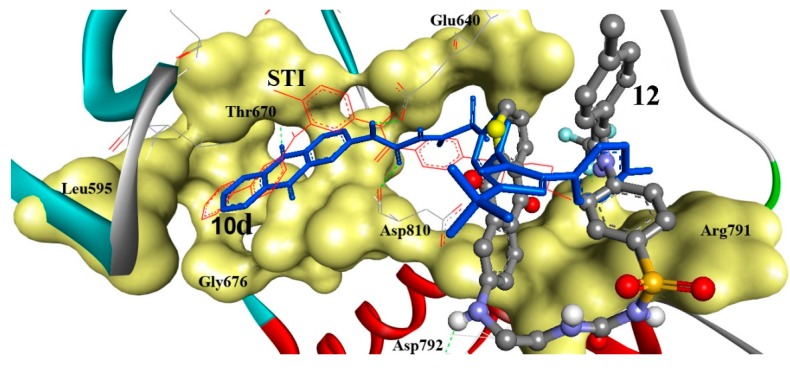
Comparative docking modes of compounds **10d** (blue sticks) and **12** (ball and stick, colored by elements; grey = carbon, red = oxygen, blue = nitrogen, and yellow = sulfur), involving flexible docking into 1t46, c-Kit-PTK. Compounds **10d** and **12** exhibited three and two hydrogen bonds, respectively. Compound **10d** is shown superimposed (RMSD: 4.29 Å) onto the STI ligand (red lines). Compound **12** was partially shifted from the STI ligand (RMSD: 10.84 Å). PTK is shown as a solid backbone ribbon for protein and its binding site is shown in a yellow solid surface view with labeled amino acids.

**Figure 4:**
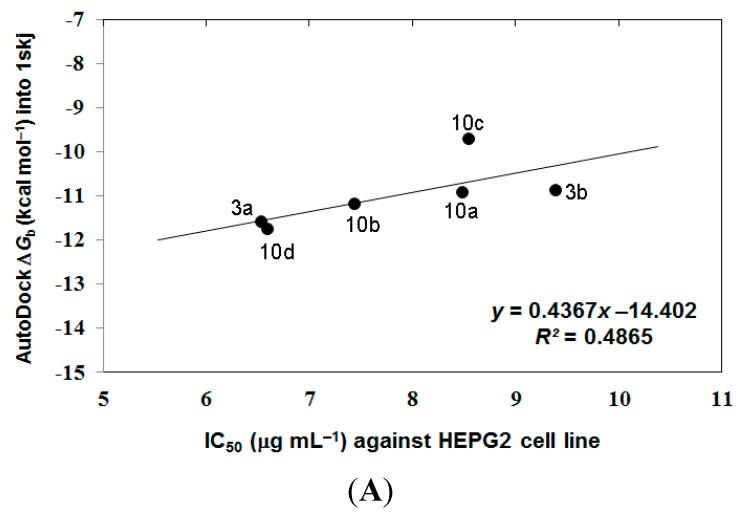

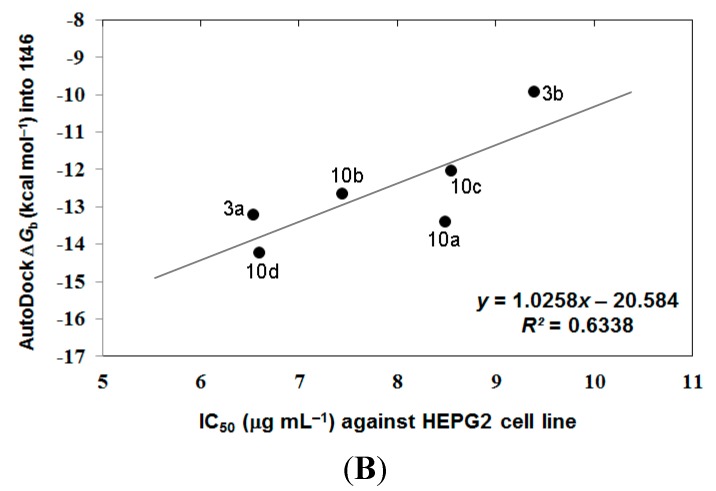
The overall correlation between IC_50_ against HEPG2 tumor cell line and the binding free energies (Δ*G*_b_) for compounds **3a**, **3b**, and **10a**–**10d** against 1skj PTK (plot **A**), and against 1t46 PTK (plot **B**).

In the analysis of docking results, a good overall correlation exists between the biological results (IC_50_ (μg·mL^−1^) against HEPG2 tumor cells) and their corresponding binding affinities predicted by AutoDock into the corresponding 1skj (Pp60v-src) tyrosine kinase. Many compounds, namely **3a**, **3b**, and **10a**–**10d**, again revealed a reasonable correlation coefficient (*R*^2^) of 0.487 as represented in Figure 4A. These compounds revealed better correlation coefficient (*R*^2^) between their biological results (IC_50_ (μg·mL^−1^) against HEPG2 tumor cells and binding free energies (∆*G*_b_) into 1t46 (PDGFR, c-Kit) tyrosine kinase, being of 0.656 as represented in Figure 4B.

Accordingly, from the aforementioned correlations between the biological activity and molecular docking results, we can conclude that our compounds in this study are proposed to act via inhibition of the c-Kit receptor PTK: Platelet-derived growth factor receptor (PDGFR, c-Kit; pdb code: 1t46) more than through the non-receptor tyrosine kinase SRC (Pp60v-src; pdb code: 1skj).

## 3. Experimental Section

### 3.1. Chemistry

Melting points were obtained on a Barnstead 9001 Electrothermal melting point apparatus (Chula Vista, CA, USA) and are uncorrected. IR spectra were recorded on a Perkin Elmer FT-IR Spectrum BX Spectrometer (Waltham, MA, USA) at cm^−1^ scale using KBr discs. ^1^H NMR and ^13^C NMR were recorded on a JEOL 300 MHz Spectrometer (Tokyo, Japan), Japan and chemical shift values were expressed in δ values (ppm) relative to tetramethylsilane (TMS) as internal standard. Coupling constants are given in Hz. The mass spectra were recorded on GCMC-QP 1000 EX Shimadzu Gas Chromatography MS spectrometer (Kyoto, Japan), Japan E.I.70 ev. Elemental analysis (C, H, N) were carried out at the Micro analytical Center, Faculty of Science, Cairo University, Cairo, Egypt, and were in full agreement with the proposed molecular weights within ± (0.2%–0.3%) of the theoretical values. All reagents were of commercial quality and were used without further purification. Organic solvents were dried in the presence of an appropriate drying agent and were stored over suitable molecular sieves. Reaction progress was monitored by analytical thin layer chromatography (TLC) on precoated (0.75 mm) silica gel GF254 plates and the products were visualized by UV light.

#### 3.1.1. *N*-2-Chloroacyl-4-[5-(*p*-tolyl)-3-trifluoromethyl-1*H*-pyrazol-1-yl] benzene sulfonamide (**2a**)

According to Reported Method [19]. *N*-(2- or 4-Chloroalkyl)-4-[5-(*p*-tolyl)-3-trifluoromethyl-1*H*-pyrazol-1-yl] Benzene Sulfonamide (**2b**,**c**). A mixture of celecoxib **1** (0.01 mol) and 1,2-chloropropionyl chloride, or chlorobutyryl chloride (0.04 mol) was heated under reflux for 1 h, the mixture was concentrated by evaporation under vacuum, after cooling the reaction mixture was poured onto ice cold water and the solid formed was filtered off, dried and recrystallized from ethanol to give the compounds **2b**,**c** respectively.

#### 3.1.2. *N*-(2-Chloropropionyl)-4-[5-(*p*-tolyl)-3-trifluoromethyl-1*H*-pyrazol-1-yl] benzene sulfonamide (**2b**)

Yield 82%; m.p.: 144–146 °C; IR (ν*_max_*/cm^−1^): 3232 (NH), 1732 (C=O); ^1^H NMR (DMSO-*d*_6_) δ: 1.40 (d, *J* = 6.5 Hz, 3H, CH_3_), 2.20 (s, 3H, Ar–CH_3_), 4.40 (q, 1H, CH–Cl), 6.67 (s, 1H, pyrazole-H), 7.19–7.95 (m, 9H, Ar–H + NH); ^13^C NMR (DMSO-*d*_6_) δ: 20.6 (CH_3_), 21.3 (Ar–CH_3_), 56.5 (Cl–CH), 106.1, 125.3, 125.9, 126.7, 128.7, 129.3, 139.0, 141.0, 142.6, 143.9, 145.2, 154.5 (CF_3_, Ar–C and C=N), 175.2 (C=O); MS: *m*/*z* (%): 471, 473 (M^+^, M^+2^, 28.8, 11.2)

#### 3.1.3. *N*-(4-Chlorobutyryl)-4-[5-(*p*-tolyl)-3-trifluoromethyl-1*H*-pyrazol-1-yl] benzene sulfonamide (**2c**)

Yield 75%; m.p.: 135 °C; IR (ν*_max_*/cm^−1^): 3244 (NH), 1735 (C=O); ^1^H NMR (DMSO-*d*_6_) δ: 2.0 (m, 2H, CH_2_–CH_2_–CH_2_), 2.3 (s, 3H, Ar–CH_3_), 2.39 (t, *J* = 7.5 Hz, 2H, COCH_2_), 3.8 (t, *J* = 7.5 Hz, 2H, CH_2_Cl), 6.67 (s, 1H, pyrazole–H), 7.0 (d, *J* = 8 Hz, 2H, H-3, 5 *p*-tolyl), 7.1 (d, *J* = 8 Hz, 2H, H-2, 6 *p*-tolyl), 7.4 (d, *J* = 9 Hz, 2H, H-2, 6 benzene sulfonamide), 7.9 (d, *J* = 9 Hz, 2H, H-3, 5 benzene sulfonamide); ^13^C NMR (DMSO-*d*_6_) δ: 18.2 (CH_2_–CH_2_–CH_2_), 22.9 (CH_3_), 30.3 (CH_2_CO), 47.3 (CH_2_Cl), 106.4, 125.3, 125.6, 128.7, 129.1, 129.8, 130.8, 137.2, 139.9, 143.6, 145.3, 154.5 (CF_3_, Ar–C and C=N), 173.2 (C=O); MS: *m*/*z* (%): 485, 487 (M^+^, M^+2^, 37.8, 13.2).

#### 3.1.4. General Method for Preparation of *N*-(2- or 4-(9,10-Dihydro-9,10-dioxoanthracen-2-yl) aminoacyl) 4-[5-(*p*-tolyl)-3-trifluoromethyl-1*H*-pyrazol-1-yl] benzene sulfonamide (**3a**–**c**)

To a solution of **2a**–**c** (0.01 mol) in ethanol (15 mL), 2-aminoanthraquinone **8** (0.01 mol) in DMF (3 mL) was added. The mixture was then heated to reflux for 4–5 h, the excess solvent was removed under vacuum and after cooling the precipitate formed was collected by filtration, washed with water and recrystallized from the proper solvent to obtain **3a**–**c**, respectively.

#### 3.1.5. *N*-(2-(9,10-Dihydro-9,10-dioxoanthracen-2-yl) aminoacetyl)-4-[5-(*p*-tolyl)-3-trifluoromethyl-1*H*-pyrazol-1-yl] benzene sulfonamide (**3a**)

Yield 70%; Crystallized from ethanol; m.p.: 156–158 °C; IR (ν*_max_*/cm^−1^): 3345, 3234 (2NH), 1673 (br., 3C=O); ^1^H NMR (DMSO-*d*_6_) δ: 2.30 (s, 3H, Ar–CH_3_), 4.30 (s, 2H, N–CH_2_), 6.67 (s, 1H, pyrazole–H), 7.0–7.1 (d, d, *J* = 8 Hz, 2H, H-3, 4 anthraquione), 7.36 (s, 1H, H-1, anthra quinone), 7.4 (d, *J* = 8.5 Hz, 2H, H-3, 5 *p*-tolyl), 7.6–8.1 (m, 10 H, Ar–H), 8.9 (br. s, 2H, 2NH); ^13^C NMR (DMSO-*d*_6_) δ: 21.3 (Ar–CH_3_), 29.7 (CH_2_), 106.3, 109.6, 118.0, 121.0, 126.1, 126.3, 126.4, 126.7, 128.7, 128.9, 129.60, 133.0, 133.3, 133.7, 134.3, 134.9, 137.6, 139.2, 142.3, 154.7 (CF_3_, Ar–C and C=N), 173.9, 180.1, 183.3 (3C=O); MS: *m*/*z* (%): 644 (M^+^, 14.7).

#### 3.1.6. *N*-(2-(9,10-Dihydro-9,10-dioxoanthracen-2-yl)-aminopropionyl)-4-[5-(*p*-tolyl)-3-trifluoromethyl-1*H* pyrazol-1-yl] benzene sulfonamide (**3b**)

Yield 75%; Recrystallized from dil. ethanol; m.p.: 261–263 °C; IR (ν*_max_*/cm^−1^): 3361, 3228 (2NH), 1672, 1628 (3C=O); ^1^H NMR (DMSO-*d*_6_) δ: 1.4 (d, *J* = 6.5 Hz, 3H, CH–CH_3_), 2.2 (s, 3H, Ar–CH_3_), 3.9 (q, 1H, CH–CH_3_), 6.67 (s, 1H, pyrazole-H), 7.0–7.1 (d, d, *J* = 8 Hz, 2H, H-3, 4 anthraquinone), 7.3 (d, *J* = 8 Hz, 5, 2H, H-3, 5 *p*-tolyl), 7.4–8.1 (m, 11 H, Ar–H), 10.9 (br. s, 2H, 2-NH); ^13^C NMR (DMSO-*d*_6_) δ: 17.6 (CH_3_), 21.3 (Ar–CH_3_), 59.5 (NH–CH), 109.2, 109.6, 110.2, 116.5, 118.0, 121.1, 126.3, 126.9, 129.5, 133.0, 133.3, 133.7, 134.3, 134.9, 139.8, 141.3, 142.5, 144.6, 145.2, 154.7 (CF_3_, Ar–C and C=N), 175.2, 180.0, 183.3 (3C=O); MS: *m*/*z* (%): 658 (M^+^, 12.9).

#### 3.1.7. *N*-(4-(9,10-Dihydro-9,10-dioxoanthracen-2-yl-aminobutanoyl)-4-[5-(*p*-tolyl)-3-trifluoromethyl-1*H*-pyrazol-1-yl] benzene sulfonamide (**3c**)

Yield: 82%, Recrystallized from CHCl_3_; m.p.: 214–216 °C, IR (ν*_max_*/cm^−1^): 3359, 3227 (2NH), 1672 (br, 3C=O), ^1^H NMR (DMSO-*d*_6_) δ: 1.20 (m, 2H, –CH_2_–CH_2_–CH_2_), 2.02 (t, 2H, CH_2_–CO), 2.30 (s, 3H, Ar–CH_3_), 3.80 (t, 2H, N–CH_2_), 6.67 (s, 1H, pyrazole–H), 7.02–7.1 (d, d, *J* = 8 Hz, 2H, H-3, 4 anthra quione), 7.2 (s, 1H, H-1 anthraquinone), 7.3 (d, *J* = 8.5 Hz, 2H, H-3, 5 *p*-tolyl), 7.5 (d, *J* = 9 Hz, 2H, H-2, 6 *p*-tolyl), 7.8–8.1 (m, 8H, Ar–H), 10.1, (br. s, 2H, 2-NH); ^13^C-NMR (DMSO-*d*_6_) δ: 17.8 (CH_2_–CH_2_–CH_2_), 20.7 (Ar–CH_3_), 31.6 (–CH_2_CO), 47.3 (N–CH_2_), 106.3, 109.6, 118.0, 121.0, 126.1, 126.3, 126.4, 126.7, 128.7, 128.9, 129.4, 129.6, 133.0, 133.3, 133.7, 134.3, 134.9, 137.6, 139.2, 142.3, 154.7 (CF_3_, Ar–C and C=N), 173.9, 180.1, 183.3 (3C=O); MS: *m*/*z* (%): 672 (M^+^, 48.1).

#### 3.1.8. *N*-(2-Isocyanatoethyl)-4-[5-(*p*-tolyl)-3-trifluoromethyl-1*H*-pyrazol-1-yl] benzene sulfonamide (**4**)

A mixture of **1** (0.01 mol) and 2-chloroethyl isocyanate (0.04 mol) was heated under reflux for 1 h. The excess 2-chloroethylisocyanate was removed under vacuum, after cooling, the reaction mixture was poured into ice cold water the solid formed was filtered off, washed with water and recrystallized from ethanol to give compound **4** in 85% yield; m.p.: 115–117 °C; IR (ν*_max_*/cm^−1^), 3321 (NH), 1740 (C=O); ^1^H NMR (CDCl_3_) δ: 2.30 (s, 3H, CH_3_–Ar), 3.60 (t, *J* = 6.5 Hz, 2H, CH_2_), 4.2 (t, *J* = 6.5 Hz, 2H, CH_2_), 6.67 (s, 1H, pyrazole-H), 7.09 (d, *J* = 10 Hz, 2H, H-3, 5 *p*-tolyl), 7.18 (d, *J* = 11 Hz, 2H, H-2, 6 *p*-tolyl), 7.4–8.03 (m, 5H, Ar–H and NH); ^13^C NMR (CDCl_3_) δ: 20.2 (CH_3_), 39.9, 41.8, (2CH_2_), 105.5, 124.1, 124.2, 127.4, 128.0, 128.5, 134.9, 138.7, 142.7, 144.1, 147.3, 150.5, 150.9 (CF_3_, Ar–C, C=N and N=C=O); MS: *m*/*z* (%): 450 (M^+^, 10.5).

#### 3.1.9. *N*-(2-(3-(9,10-Dihydro-9,10-dioxoanthracen-2-yl) ureido) ethyl)-4-(5-(*p*-tolyl)-3-(trifluoro methyl)-1*H*-pyrazol-1-yl) benzene sulfonamide (**5**)

To a solution of compound **4** (0.01 mol) in ethanol (15 mL), 2-aminoanthraquinone **8** (0.01 mol) in DMF (3 mL) was added. The mixture was then heated to reflux for 5 h. the excess solvent was removed under vacuum and after cooling the precipitate formed was collected by filtration, washed with water and recrystallized from ethanol to give compound **5** in 68% yield; m.p.: 221–223 °C; IR (ν*_max_*/cm^−1^): 3435, 3345, 3200 (3NH), br. 1673 (3C=O); ^1^H NMR (DMSO-*d*_6_) δ: 2.3 (s, 3H, Ar–CH_3_), 3.7(t, *J* = 6.5 Hz, 2H, CH_2_–NHSO_2_–), 3.96 (t, *J* = 6.5 Hz, 2H, CH_2_–NH–C=O), 6.67 (s, 1H, pyrazole-H), 6.94–6.96 (d, d, *J* = 8.5 Hz, 2H, H-3, 4 anthraquinone), 7.3 (d, *J* = 8.8 Hz, 2H, H-3, 5 *p*-tolyl), 7.6 (d, *J* = 9 Hz, 2H, H-2, 6 *p*-tolyl), 7.84–8.08 (m, 9H, Ar–H), 8.25 (br. s, 3H, 3NH); ^13^C NMR (DMSO-*d*_6_) δ: 20.7 (CH_3_), 41.3, 43.2 (2C, 2CH_2_), 109.6, 118.0, 121.0, 125.1, 126.2, 126.3, 126.4, 128.7, 129.2, 129.4, 129.6, 133.0, 133.3, 133.7, 134.3, 134.8, 136.8, 139.1, 143.1, 145.4, 151.4, 151.8 (CF_3_, Ar–C and C=N), 154.7, 180.0, 183.3 (3C=O); MS: *m*/*z* (%): 673 (M^+^, 3.75).

#### 3.1.10. *N*-[2-(2-Chloroethylamino) ethyl]-4-[5-(*p*-tolyl)-3-trifluoromethyl-1*H*-pyrazol-1-yl] benzene sulfonamide (**6**)

Bis (2-chloroethyl) amine hydrochloride (0.04 mol) was added to a solution of celecoxib **1** (0.02 mol) in dry toluene (10 mL) within 5 min in an ice bath and triethylamine (5 drops) was added and the reaction mixture was refluxed for 4 h. After the reaction was completed the solvent was concentrated, the residue was cooled and poured onto ice-cold water. The reaction mixture was then left at 5 °C overnight. The solid formed was collected by filtration and recrystallized from ethanol to give compound **6** in 84% yield; m.p.: 156–158 °C; IR (ν*_max_*/cm^−1^): 3340, 3234 (2NH), ^1^H NMR (DMSO-*d*_6_) δ: 2.2 (s, 3H, Ar–CH_3_), 2.5–2.55 (m, 6H, 3CH_2_NH), 3.8 (t, *J* = 6.5 Hz, 2H, CH_2_Cl), 6.7 (s, 1H, pyrazole-H), 7.10 (d, *J* = 8 Hz, 2H, H-3, 5 *p*-tolyl), 7.19 (d, *J* = 8 Hz, 2H, H-2, 6 *p*-tolyl), 7.5 (d, *J* = 8.8 Hz, 2H, H2, 6 benzene sulfonamide), 8.1 (d, *J* = 8.7 Hz, 2H, H-3, 5 benzene sulfonamide), 8.7 (s, 1H, NH), 11.02 (s, 1H, NH); ^13^C NMR (DMSO-*d*_6_) δ: 20.2 (Ar-CH_3_), 39.5, 40.0, 40.7, 42.0 (4 CH_2_), 105.5, 124.2, 124.4, 127.5, 128.2, 128.7, 135.0, 138.8, 142.8, 144.2, 150.6, 151.1 (CF_3_, Ar–C and C=N); MS: *m*/*z* (%): 523, 525 (M^+^, M^+2^, 75.6, 27).

#### 3.1.11. *N*-[2-(2-(9,10-Dihydro-9,10-dioxoanthracen-2-yl) aminoethyl) aminoethyl]-4-[5-(*p*-tolyl)-3-trifluoromethyl-1*H*-pyrazol-1-yl] benzene sulfonamide (**7**)

A mixture of **6** (0.01 mol) in ethanol (15 mL), and 2-aminoanthraquinone **8** (0.01 mol) in DMF (3 mL) was heated under reflux for 8 h. The excess solvent was evaporated under vacuum and after cooling the solid formed was filtered off, washed well with water and recrystallized from methanol to give compound **7** in 73% yield; m.p.: 157–159 °C; IR (ν*_max_*/cm^−1^): 3415, 3351, 3234 (3NH), 1672 (2C=O), 1618 (C=C); ^1^H NMR (DMSO-*d*_6_) δ: 2.2 (s, 3H, Ar–CH_3_), 2.53–2.64 (m, 8H, 4CH_2_), 6.67 (s, 1H, pyrazole-H), 6.94–6.96 (d, d, *J* = 8.5 Hz, 2H, H-3,4 anthraquinone), 7.0 (d, *J* = 8 Hz, 2H, H-3, 5 *p*-tolyl), 7.1 (d, *J* = 8.8 Hz, 2H, H-2, 6 *p*-tolyl), 7.2–8.1 (m, 11H, Ar–H and 2NH); 8.3 (br. s, 1H, NH), ^13^C NMR (DMSO-*d*_6_) δ: 20.3 (Ar–CH_3_), 39.6, 40.1, 40.9, 42.2 (4CH_2_), 105.5, 117.2, 124.2, 124.4, 125.1, 126.6, 126.7, 127.5, 128.3, 128.9, 129.2, 129.6, 133.0, 134.0, 134.5, 135.0, 137.8, 138.6, 142.8, 143.1, 144.2, 150.6 (CF_3_, Ar–C and C=N), 173.8, 180.1, 183.3 (3CO); MS: *m*/*z* (%): 709, 711 (M^+^, M^+2^, 68.8, 27).

#### 3.1.12. General Method for Preparation of 2- or 4-Chloro-*N*-(9,10-dihydro-9,10-dioxoanthracen-6-yl) alkanamide (**9b**,**c**)

A mixture of anthraquinone 8 (0.01 mol) and 1,2-chloropropionyl chloride, or chlorobutyryl chloride (0.04 mol) was heated under reflux for 1 h, the mixture was concentrated by evaporation under vacuum, after cooling the reaction mixture was poured onto ice cold water and the solid formed was filtered off, dried and recrystallized from ethanol to give the compounds **9b**,**c** respectively.

#### 3.1.13. 2-Chloro-*N*-(9,10-dihydro-9,10-dioxoanthracen-6-yl) propanamide (**9b**)

Yield 78%; Crystallized from ethanol; m.p.: 168 °C; IR (ν*_max_*/cm^−1^): 3348 (NH), 1716, 1671 (3C=O); ^1^H NMR (DMSO-*d*_6_) δ: 2.9 (d, *J* = 7.5 Hz, 3H, CH_3_), 5.5 (q, 1H, CHCl), 8.3 (s, 1H, H-1 anthraquinone), 9–9.7 (m, 6 H, Ar–H), 10.2 (s, 1H, NH); ^13^C NMR (DMSO-*d*_6_) δ: 17.2 (CH_3_), 50.7 (CH), 111.9, 119.2, 122.0, 124.0, 124.6, 128.2, 128.3, 128.7, 129.0, 129.4, 137.1, (Ar–C), 162.6 (CO), 176.7, 177.4 (2CO anthraquinone); MS: *m*/*z* (%): 313, 315 (M^+^, M^+2^, 34.4, 13.5).

#### 3.1.14. 4-Chloro-*N*-(9,10-dihydro-9,10-dioxoanthracen-6-yl) butanamide (**9c**)

Yield 75 %; Crystallized from dilute ethanol; m.p.: 219 °C; IR (ν*_max_*/cm^−1^): 3340 (NH), 1698, 1667, 1649 (3C=O); ^1^H NMR (DMSO-*d*_6_) δ: 2.0 (m, 2H, CH_2_–CH_2_–CH_2_), 2.5 (t, *J* = 6.5 Hz, 2H, COCH_2_), 3.7 (t, *J* = 6.5 Hz, 2H, CH_2_Cl), 7.9–8.2 (m, 6 H, Ar–H), 8.4 (s, 1H, Ar, H-1), 10.6 (s, H, NH); ^13^C NMR (DMSO-*d*_6_) δ: 27.5 (CH_2_–CH_2_–CH_2_), 33.5 (COCH_2_), 62.9 (CH_2_Cl), 115.7, 123.6, 126.6, 126.7, 127.7, 128.4, 133.0, 134.1, 134.19, 134.5, 144.6 (Ar–C), 171 (C=O), 181.3, 182.4 (2C=O anthraquinone); MS: *m*/*z* (%): 327, 329 (M^+^, M^+2^, 28.4, 12.5).

#### 3.1.15. *N*-(9,10-Dihydro-9,10-dioxoanthracen-2-yl-amino-carbonyl)-4-[5-(*p*-tolyl)-3-trifluoromethyl-1*H*-pyrazol-1-yl] benzene sulfonamide derivatives (**10a**–**d**)

General method: A mixture of **9a**–**d** (0.01 mol) in ethanol (15 mL), and celecoxib **1** (0.01 mol) in DMF (3 mL) was heated under reflux for 4–5 h. The excess solvent was evaporated under vacuum and the solid formed was filtered off, washed with water and recrystallized from the proper solvent to give compounds **10a**–**d**.

#### 3.1.16. *N*-(9,10-Dihydro-9,10-dioxoanthracene-2-yl) aminoacetyl)-4-[5-(*p*-tolyl)-3-trifluoromethyl-1*H*-pyrazol-1-yl] benzene sulfonamide (**10a**)

Yield 70%; Recrystallized from chloroform; m.p.: 245–247 °C; IR (ν*_max_*/cm^−1^): 3345, 3114, (2NH), br. 1697 (3C=O); ^1^H NMR (DMSO-*d*_6_) δ: 2.5 (s, 3H, Ar–CH_3_), 4.37 (s, 2H, CH_2_), 6.68 (s, 1H, pyrazole-H), 6.94–6.96 (d, d, *J* = 8.5 Hz, 2H, H-3, 4 anthraquinone), 7.3 (d, *J* = 8 Hz, 2H, H-3, 5 *p*-tolyl), 7.6 (d, *J* = 8.5 Hz, 2H, H-2, 6 *p*-tolyl), 7.84–8.1 (m, 9H, Ar–H) 8.45 (s,1H, NH), 10.9 (s, 1H, NH); ^13^C NMR (DMSO-*d*_6_) δ: 21.3 (CH_3_), 43.0 (CH_2_), 106.3, 109.2, 109.6, 110.2, 116.5, 117.2, 118.0, 121.9, 125.1, 126.6, 126.7, 128.2, 129.6, 134.0, 134.3, 134.5, 143.9, 144.6, 145.2, 157.5 (CF3, Ar–C and C=N), 167.5, 181.3, 182.3 (3CO); MS: *m*/*z* (%): 644 (M^+^, 61.7).

#### 3.1.17. *N*-[1-(9,10-Dihydro-9,10-dioxoanthracene-2-yl) aminocarbonyl) ethyl]-4-[5-(*p*-tolyl)-3-tri fluoromethyl-1*H*-pyrazol-1-yl] benzene sulfonamide (**10b**)

Yield 80%; Recrystallized from dil. ethanol; m.p.:132–135 °C; IR(ν*_max_*/cm^−1^): 3346, 3200 (2NH), 1690, 1673 (3C=O); ^1^H NMR (DMSO-*d*_6_) δ: 1.6 (d, *J* = 6.5 Hz, 3H, CH_3_), 2.28 (s, 3H, Ar–CH_3_), 4.7 (q, 1H, CH–CH_3_), 6.67 (s, 1H, pyrazol-H), 6.94–6.96 (d, d, *J* = 8.5 Hz, 2H, H-3, 4 anthraquinone), 7.3 (d, *J* = 8 Hz, 2H, H-3, 5 *p*-tolyl), 7.6 (d, *J* = 8.5 Hz, 2H, H-2, 6 *p*-tolyl), 7.84–8.1 (m, 9H, Ar–H) 8.46 (s, 1H, NH), 10.99 (s, 1H, NH); ^13^C NMR (DMSO-*d*_6_) δ: 16.5 (CH_3_), 20.7 (CH_3_–Ar), 52.7 (CH), 106.3, 109.6, 118.0, 121.9, 126.1, 126.3, 126.7, 128.7, 128.9, 129.6, 133.0, 133.3, 133.7, 134.3, 134.9, 137.6, 139.2, 142.3, 154.7 (CF_3_, Ar–C and C=N), 173.9, 180.1, 183.3 (3C=O); MS: *m*/*z* (%): 658 (M^+^, 30).

#### 3.1.18. *N*-[3-(9,10-Dihydro-9,10-dioxoanthracene-2-yl) aminocarbonyl) propyl]-4-[5-(*p*-tolyl)-3-tri fluoromethyl-1*H*-pyrazol-1-yl] benzene sulfonamide (**10c**)

Yield 78%; Recrystallized from ethanol; m.p.: 113–115 °C; IR (ν*_max_*/cm^−1^): 3343, 3200 (2NH), 1690, 1673 (3C=O); ^1^H NMR (DMSO-*d*_6_) δ: 1.96 (m, 2H, CH_2_CH_2_CH_2_), 2.1 (t, *J* = 6.5 Hz, 2H, CH_2_CO) 2.2 (s, 3H, CH_3_–Ar), 2.6 (t, *J* = 6.5 Hz, 2H, N–CH_2_), 6.6 (s, 1H, pyrazole-H), 6.94–6.96 (d, d, *J* = 8.5 Hz, 2H, H-3, 4 anthraquinone), 7.3 (d, *J* = 2 Hz, 2H, H-3, 5 *p*-tolyl), 7.6 (d, *J* = 9 Hz, 2H, H-2, 6 *p*-tolyl), 7.84–8.1 (m, 9H, Ar-H), 8.4 (s, 1H, NH), 8.46 (s, 1H, NH); ^13^C NMR (DMSO-*d*_6_) δ: 21.3 (CH_3_), 29.7 (CH_2_–CH_2_–CH_2_), 32.8 (CH_2_CO), 48.5 (CH_2_-N), 106.3, 115.7, 124.7, 125.4, 125.7, 127.2, 127.5, 128.7, 129.7, 133.5, 133.6, 133.9, 134.2, 139.8, 141.3, 142.5, 144.6, 145.2, (CF_3_, Ar–C and C=N), 175.0, 180.0, 183.3 (3C=O); MS: *m*/*z* (%): 672 (M^+^, 46.6).

#### 3.1.19. *N*-(9,10-Dihydro-9,10-dioxoanthracene-2-yl)aminooxalyl)-4-[5-(*p*-tolyl)-3-trifluoromethyl-1*H*-pyrazol-1-yl] benzene sulfonamide (**10d**)

Yield 72%; Recrystallized from chloroform/pet. ether; m.p.: 132–135 °C; IR (ν*_max_*/cm^−1^): 3415, 3245 (2NH), 1728, 1620 (4 C=O); ^1^H NMR (DMSO-*d*_6_) δ: 2.2 (s, 3H, Ar–CH_3_), 6.69 (s, 1H, pyrazole-H), 7.2 (d, *J* = 8 Hz, 2H, H-3, 5 *p*-tolyl), 7.5–8.1 (m, 13H, Ar-H), 8.7 (s, 1H, NH), 11.02 (s, 1H, NH); ^13^C NMR (DMSO-*d*_6_) δ: 106.3, 109.60, 110.2, 116.5, 117.2, 118.0, 121.9, 125.1, 126.6, 126.7, 128.2, 129.6, 133.0, 134.0, 134.3, 134.5, 143.9, 144.6, 145.2, 157.5 (CF_3_, Ar–C and C=N), 173.2, 180.1, 183.3 (4C=O); MS: *m*/*z* (%): 658 (M^+^, 44).

#### 3.1.20. 2-(2-Isocyanatoethylamino)anthracene-9,10-dione (**11**)

A mixture of **8** (0.01 mol) and 2-chloroethyl isocyanate (0.04 mol) was heated under reflux for 1 h. The excess 2-chloroethylisocyanate was removed under vacuum, after cooling, the reaction mixture was poured onto ice cold water the solid formed was filtered off, washed with water and recrystallized from ethanol to give compound **11** in 75% yield; m.p.: 144 °C; IR (ν*_max_*/cm^−1^): 3343 (NH), 1735, 1708, 1668 (3C=O); ^1^H NMR (DMSO-*d*_6_) δ: 4.1 (t, *J* = 7.5 Hz, 2H, CH_2_NCO), 3.9 (t, *J* = 7.5 Hz, 2H, HN–CH_2_), 7.9–8.1 (m, 6H, Ar–H), 8.4 (s, 1H, Ar, H-1), 8.9 (s, 1H, NH); ^13^C NMR (DMSO-*d*_6_) δ: 49.9 (CH_2_), 58.7 (CH_2_), 115.7, 123.6, 126.6, 126.7, 127.7, 128.4, 133.0, 134.1, 134.19, 134.5, 144.6 (Ar–C), 173.9 (N=C=O), 181.3, 182.4 (2C=O anthraquinone); MS: *m*/*z* (%): 292 (M^+^, 14.5).

#### 3.1.21. *N*-[2-(9,10-Dihydro-9,10-dioxoanthracene-2-yl-amino) ethylcarbamoyl]-4-[5-(*p*-tolyl)-3-trifluoromethyl-1*H*-pyrazol-1-yl] benzene sulfonamide (**12**)

A mixture of **11** (0.01 mol) in ethanol (15 mL), and celecoxib **1** (0.01 mol) in DMF (3 mL) was heated under reflux for 5 h. The excess solvent was evaporated under vacuum and after cooling the solid formed was filtered off, washed with water and recrystallized. Yield 75%; crystallized from dil. ethanol; m.p.: 162–164 °C; IR (ν*_max_*/cm^−1^): 3306, 3220 (3NH), 1671 (br., 3C=O); ^1^H NMR (DMSO-*d*_6_) δ: 2.2 (s, 3H, Ar-CH_3_), 3.4 (m, 2H, CH_2_), 4.1 (m, 2H, CH_2_), 6.6 (s, 1H, pyrazole-H), 7.02–7.1 (d, d, *J* = 8 Hz, 2H, H-3, 4 anthraquione), 7.2 (s, 1H, H-1, anthra quinone), 7.3 (d, *J* = 8.5 Hz, 2H, H-3, 5 *p*-toplyl), 7.5 (d, *J* = 9 Hz, 2H, H-2, 6 *p*-tolyl), 7.8–8.1 (m, 8H, Ar-H), 9.48 (s, 1H, NH), 10.09 (s, 1H, NH), 10.4 (s, 1H, NH); ^13^C NMR (DMSO-*d*_6_) δ: 20.7 (Ar–CH_3_), 43.8, 50.5 (2CH_2_), 106.3, 115.7, 123.5, 124.7, 125.4, 125.9, 126.5, 126.7, 127.2, 127.5, 128.7, 129.3, 133.5, 134.0, 139.8, 141.3, 142.5, 144.6, 145.2 (CF_3_, Ar–C and C=N), 175.2, 181.2, 183.3 (3C=O); MS: *m*/*z* (%): 673 (M^+^, 42).

### 3.2. Pharmacological Screening

#### 3.2.1. Measurement of Potential Cytotoxicity by Sulforhodamine B (SRB) Assay

The selected derivatives (compounds **3a**–**c**, **7**, **10a**–**d** and **12**), were subjected to a screening system for evaluation of their antitumor activity against liver HEPG2 cancer cell lines in comparison to the known anticancer drugs: 5-FU and DOX. Potential cytotoxicity of the compounds in this study was investigated using the method of Skehan *et al*. [27]*.* Cells were plated in 96-multiwell plate (10^4^ cells/well) for 24 h before treatment with the compounds to allow attachment of cells to the wall of the plate. Different concentrations of the compound under test (0, 1, 2.5, 5, 10 µg/mL) were added to the cell monolayer. Triplicate wells were prepared for each individual dose. Monolayer cells were incubated with the compounds for 48 h at 37 °C and in an atmosphere of 5% CO_2_. Cultures were then fixed with trichloroacetic acid and stained for 30 min with 0.4% (*w*/*v*) sulforhodamine B (SRB) dissolved in 1% acetic acid. Unbound dye was removed by four washes with 1% acetic acid, and protein-bound dye was extracted with 10 mM unbuffered tris base (tris hydroxymethyl aminomethane, Meryer Chemical Technology Co., Shanghai, China) for determination of optical density in a computer-interfaced, 96-well micro titer plate reader. The relation between surviving fraction and drug concentration is plotted to get the survival curve of both cancer cell lines after the specified compound.

#### 3.2.2. Biochemical Analysis

Male albino mice weighing 18–20 g were used in the present study. Mice were divided into three main groups as follows: Untreated or control group (5 mice each), the second group is, divided into two subgroups (5 mice for each subgroup) and treated with 5-FU or DOX as reference anticancer drugs and the third group is divided into nine subgroups (5 mice for each subgroup) which was treated with the selected derivatives. In the control group each mouse was given a single inter peritoneal i.p. injection of 0.1 mL DMSO while the second and the third groups were given a single i.p. injection of 0.1 mL containing 12 mg/kg body weight of the standard or tested compounds. 5-FU or DOX was dissolved in sterile water and the synthesized compounds were dissolved in DMSO. Blood was collected after 7 days from all mice groups. The biochemical effects of the selected compounds, on some liver enzymes such as aspartate, alanine aminotransferases (AST and ALT) [28] and alkaline phosphatase (ALP) [29], were analyzed using a blood auto analyzer (Olympus AV 400, Tokyo, Japan). Moreover, albumin [30], globulins [31], creatinine [32], total lipids [33], cholesterol [34], triglycerides and bilirubin [35] in serum of mice were evaluated in comparison to 5-FU and DOX. Statistical analysis of the results was performed using Chi-square values (SPSS computer program, IBM Corporation, NY, USA).

### 3.3. Molecular Docking

This protocol presents a detailed outline and advice for use of AutoDock (Molecular Graphics Lab., The Scripps Research Institute, La Jolla, CA, USA) and its graphical interface, AutoDock Tools, to analyze biomolecular complexes using computational docking. The first step is to prepare the coordinate files for the docking molecule and the target molecule. The second step is the calculation of the affinity grid for the target molecule. In the third step, the docking molecule is docked with the affinity grid, and, finally, the results are analyzed.

#### 3.3.1. Preparing the Target Macromolecules Investigated

Two different target protein tyrosine kinases were investigated. These include sarcoma proto-oncogenic kinase SRC (Pp60v-src) and platelet-derived growth factor receptor (PDGFR, c-Kit. Those were retrieved from the Protein Data Bank, http://www.rcsb.org/pdb/home/home.do. For each docking target, crucial amino acids of the active site were identified using data in PDBsum, http://www.ebi.ac.uk/pdbsum/.

#### 3.3.2. Preparing a Ligand File for AutoDock

The ligands are originally drawn with a widely used chemical structure drawing software. The three-dimensional structures of the aforementioned compounds were constructed using Chem3D Ultra 8.0 software (Chemical Structure Drawing Standard; Cambridge Soft Corporation, Cambridge, MA, USA, 2003) to obtain standard 3D structures (pdb format), then they were energetically minimized by using MOPAC with 100 iterations and minimum Root Mean Square (RMS) gradient of 0.10. It is recommended to confirm whether all hydrogen atoms are in the file before working with ADT. After opening the ligand, it can be visualized and ADT now automatically computes Gasteiger charges (empirical atomic partial charges) and distinguishes between hybridization state and type of each atom. As a part of preparation, the program determines rotatable bonds of the ligand to be able to generate different conformers for the docking.

#### 3.3.3. Setting the GRID Box, Preparing the GRID Parameter File, Running AutoGrid4, and Preparation of the Flexible Residue File

The grid parameter file tells AutoGrid4 which receptor to compute the potentials around, the types of maps to compute and the location and extent of those maps. In general, one map is calculated for each atom type in the ligand plus an electrostatics map and a separate desolvation map. The types of maps depend on the types of atoms in the ligand. Thus one way to specify the types of maps is by choosing a ligand. The grid maps of 60 × 60 × 60 grid points, centered on the ligands of the complex structures, were used to cover the binding pockets. A spacing of 0.375 Å was set centered at −6.963, 60.886, and −9.54 Å, respectively for 1skj that encompassed the active site where the co-crystallized ligand; UR2: (4-[3-Carboxymethyl-3-(4-phosphonooxybenzyl)ureido]-4-[(3-cyclohexylpropyl)methylcarbamoyl] butyric acid), was embedded, was used to guide the docked inhibitors within PTK receptor, and flexible residues: GLU35, THR36, and ARG32. Whereas, The STI-571 ligand (Imatinib or Gleevec), 4-(4-methylpiperazin-1-ylmethyl)-*N*-[4-methyl-3-(4-pyridin-3-ylpyrimidin-2-ylamino)phenyl]benzamide, was centered into its c-Kit receptor PTK (pdb code: 1t46) at 27.696, 26.657, and 39.342 Å, respectively, that encompassed the active site where the co-crystallized ligand; STI-571, was embedded, and flexible residues: GLU640, THR670, and ASP810 were selected as key amino acids. Once those parameters were set in one file, AutoGrid calculates grid parameter files for each type of atom within a given area.

#### 3.3.4. Preparing the Docking Parameter File and Running AutoDock4

The docking parameter file indicates AutoDock which map files to use, the ligand molecule to move, what its center and number of torsions are, where to start the ligand, the flexible residues to move where side chain motion in the receptor is to be modeled, which docking algorithm to use and how many runs to do. It usually has the file extension, “.dpf”. Four different docking algorithms are currently available in AutoDock: SA, the original Monte Carlo simulated annealing; GA, a traditional Darwinian genetic algorithm; LS, local search; and GALS (Larmarckian genetic algorithm), which is a hybrid genetic algorithm with local search. Each search method has its own set of parameters, and these must be set before running the docking experiment itself. These parameters include what kind of random number generator to use, step sizes, *etc.* The most important parameters affect how long each docking will run. In GALS, the number of energy evaluations and the number of generations affect how long a docking will run. ADT lets you change all of these parameters, and others not mentioned here. In our study, we used AutoDock 4.2 to check the binding conformations of the synthesized compounds into different PTKs more accurately. Lamarckian genetic algorithm was used for all molecular docking simulations. Population size of 300, mutation rate of 0.02, and crossover rate of 0.8 were set as the parameters. Simulations were performed using up to 2.5 million energy evaluations with a maximum of 27,000 generations. Each simulation was performed 10 times, yielding 10 docked conformations. The lowest energy conformations were regarded as the binding conformations between the ligands and the proteins.

### 3.4. Analyzing the Docking Results

Reading a docking log or a set of docking logs is the first step in analyzing the results of docking experiments. During its automated docking procedure, AutoDock outputs a detailed record to the result file has the extension “.dlg”. The output includes many details about the docking which are output as AutoDock parses the input files and reports what it finds. After completing the runs, AutoDock begins an analysis phase and records details of that process. At the very end, it reports a summary of the amount of time taken and the words “Successful Completion”. The key results in a docking log are the docked structures found at the end of each run, the energies of these docked structures and their similarities to each other. The similarity of docked structures is measured by computing the root-mean-square-deviation, RMSD, between the coordinates of the atoms. The docking results consist of the Protein Data Bank with Change (Q) and Torsions (T) (PDBQT) of the Cartesian coordinates of the atoms in the docked molecule, along with the state variables that describe this docked conformation and position. As a result of AutoDock calculations we obtain the output file with, in our case, ten conformers of the protein-ligand complex with flexible residues and the ligand located within the binding pocket. Each structure is scored and ranked by the program using the calculated interaction energy.

## 4. Conclusions

In this study, we have identified anthraquinone derivatives linked to celecoxib as a novel class of compound with anti-proliferative activity. Some of the new compounds (**3c**, **7**, and **12**) were the most potent in the biological assay employed (e.g., produced growth inhibition potential as compared to the reference anticancer drugs with no significant difference in biochemical parameters). These experimental findings may provide support for the use of these novel compounds as new weapons in the fight against different types of cancer.

## References

[1] Aragon-Ching B.J., Dahut W.L. (2009). Cancer treatment anti-angiogenesis approach to genitourinary. Update Cancer Ther..

[2] Koki A.T., Masferrer J.L. (2002). Celecoxib: A specific Cox-2 inhibitor with anticancer properties. Cancer Control.

[3] Bamba H., Ota S., Kato A., Kawamoto C., Fujiwara K., Matsuzaki F. (2000). Effect of prostaglandin E1 on vascular endothelial growth factor production by human macrophages and colon cancer cells. J. Exp. Clin. Cancer Res..

[4] Vane J.R., Bakhle Y.S., Botting R.M. (1998). Cyclooxygenases 1 and 2. Annu. Rev. Pharmacol. Toxicol..

[5] Lrie T., Sujii M.T., Tsuji S., Yoshio T., Lshii S., Shinzaki S., Egawa S., Kakiuchi Y., Nishida T., Yasumaru M. (2007). Synergistic antitumor effects of celecoxib with 5-fluorouracil depend on IFN-γ. Int. J. Cancer.

[6] Saldivar J.S., Lopez D., Feldman R.A., Tharappel R.J., de-la Rosa A., Terreros D., Baldwin W.S. (2007). Cox-2 overexpression as a biomarker of early cervical carcinogenesis: A pilot study. Gynecol. Oncol..

[7] Dai Z., Ma X., Kang H., Gao J., Min W., Guan H., Diao Y., Lu W., Wang X. (2012). Antitumor activity of the selective cyclooxygenase-2 inhibitor, celecoxib, on breast cancer *in vitro* and *in vivo*. Cancer Cell Int..

[8] Park W., Oh T., Han J., Pyo H. (2008). Antitumor enhancement of celecoxib, a selective cyclooxygenase-2 inhibitor, in a Lewis lung carcinoma expressing cyclooxygenase-2. J. Exp. Clin. Cancer Res..

[9] Huang S., Sinicrope F. (2010). Celecoxib-induced apoptosis is enhanced by ABT-737 and by inhibition of autophagy in human colorectal cancer cells. Autophagy.

[10] Victor V., Cristina B., David C., Denise O., Alejandro F., Mack B., William G. (2006). Celecoxib inhibits cellular growth, decreases Ki-67 expression and modifies apoptosis in ovarian cancer cell lines. Arch. Med. Res..

[11] Choe M., Chen Z., Klass C., Zhang X., Shin D. (2007). Enhancement of docetaxel-induced cytotoxicity by blocking epidermal growth factor receptor and cyclooxygenase-2 pathways in squamous cell carcinoma of the head and neck. Clin. Cancer Res..

[12] Taher A.T., Hegazy G.H. (2013). Synthesis of novel bis-anthraquinone derivatives and their biological evaluation as antitumor agents. Arch. Pharm. Res..

[13] Denny W., Wakelin L. (1990). Kinetics of the binding of mitoxantrone, ametantrone and analogues to DNA: Relationship with binding mode and anti-tumour activity. Anticancer Drug Des..

[14] Kaneshiro T., Morioka T., Lnamine M., Kinjo T., Arakaki J., Chiba L., Sunagawa N., Suzui M., Yoshimi N. (2006). Anthraquinone derivative emodin inhibits tumor-associated angiogenesis through inhibition of extracellular signal-regulated kinase 1/2 phosphorylation. Eur. J. Pharmcol..

[15] Wei D., Jiang X., Zhou L., Chen J., Chen Z., He C., Yang K., Liu Y., Pei J., Lai L. (2008). Discovery of multitarget inhibitors by combining molecular docking with common pharmacophore matching. J. Med. Chem..

[16] Ali H.I., Nagamatsu T., Akaho E. (2011). Structure-based drug design and AutoDock study of potential protein tyrosine kinase inhibitors. Bioinformation.

[17] Thangapandian S., John S., Sakkiah S., Lee K.W. (2011). Molecular docking and pharmacophore filtering in the discovery of dual-inhibitors for human leukotriene A4 hydrolase and leukotriene C4 synthase. J. Chem. Inf. Model..

[18] Morris G.M., Huey R., Lindstrom W., Sanner M.F., Belew R.K., Goodsell D.S., Olson A.J. (2009). AutoDock4 and AutoDockTools4: Automated docking with selective receptor flexibility. J. Comput. Chem..

[19] Qandil M.A., el-Mohtadi F.H., Tashtoush B.M. (2011). Chemical and *in vitro* enzymatic stability of newly synthesized celecoxib lipophilic and hydrophilic amide. Int. J. Pharm..

[20] Berghot M.A., Hanna M.A., Girges M.M. (1992). Synthesis and biological activity of some heterocyclic systems containing anthrquinone. Pharmazie.

[21] Loskutov V.A., Savel’ev V.A., Konstantinova A.V. (1985). Reaction of amino derivatives of 9,10-anthraquinone with oxalyl chloride. Izvestiya Sibirskogo Otdeleniya Akademii Nauk SSSR, Seriya Khimicheskikh Nauk.

[22] Pautus S., Yee S.W., Jayne M., Coogan M.P., Simons C. (2006). Synthesis and CYP26A1 inhibitory activity of 1-(benzofuran-2-yl-(4-alkyl/aryl-phenyl)-methyl)-1H-triazoles. Bioorg. Med. Chem..

[23] Hayakawa I., Shioya R., Agatsuma T., Furukawa H., Naruto S., Sugano Y. (2004). A library synthesis of 4-hydroxy-3-methyl-6-phenylbenzofuran-2-carboxylic acid ethyl ester derivatives as anti-tumor agents. Bioorg. Med. Chem. Lett..

[24] Attoub S., Rivat C., Rodrigues S., van B.S., Bedin M., Bruyneel E., Louvet C., Kornprobst M., Andre T., Mareel M. (2002). The c-Kit tyrosine kinase inhibitor STI571 for colorectal cancer therapy. Cancer Res..

[25] Plummer M.S., Holland D.R., Shahripour A.E., Lunney A., Fergus J.H., Marks J.S., McConnell P.W., Mueller T., Sawyer T.K. (1997). Design, synthesis, and cocrystal structure of a nonpeptide Src SH2 domain ligand. J. Med. Chem..

[26] Mol C.D., Dougan D.R., Schneider T.R., Skene R.J., Kraus M.L., Scheibe D.N., Snell G.P., Zou H., Sang B.C., Wilson K.P. (2004). Structural basis for the autoinhibition and STI-571 inhibition of c-Kit tyrosine kinase. J. Biol. Chem..

[27] Skehan P., Storeng R., Scudiero D., Anne Monks A., McMahon J., Vistica D., Warren J., Bokesch H., Kenney S., Boyd M. (1990). New colorimetric cytotoxicity assay for anticancer-drug screening. J. Natl. Cancer Inst..

[28] Abo-Ghalia M.H., Soliman A.M. (2000). Synthesis and study of the antischistosomal potency and induced biological parameters of a new 2-palmitoyl analogue of the universal antihelminthic praziquantel. Acta Pol. Pharm. Drug Res..

[29] Spencer K., Price C.P. (1979). Kinetic immunoturbidimetry: The estimation of albumin. Clin. Chim. Acta.

[30] Mays A. (1969). Baseline hematological and blood biochemical parameters of the Mongolian gerbil (*Meriones unguiculatus*). Lab. Anim. Sci..

[31] Joseph V. (1999). Raptor hematology and chemistry evaluation. Vet. Clin. N. Am. Exot. Anim. Pract..

[32] Soliman A.M., Faddah L.M. (1994). Screening of two pyrozolpyrimidines schistosomicidal activity and their effect on serum transaminases of albino mice. Egypt. J. Bilh..

[33] Garde A.H., Hansen A.M., Skovgaard L.T., Christensen J.M. (2000). Seasonal and biological variation of blood concentrations of total cholesterol, dehydroepiandrosterone sulfate, hemoglobin A(1c), IgA, prolactin, and free testosterone in healthy women. Clin. Chem..

[34] Rietz E.B., Guilbault G.G. (1977). Fluorometric estimation of triglycerides in serum by a modification of the method of Bucolo and David. Clin. Chem..

[35] Guilbaud N.L., Kraus-Berthier F., Meyer-Losic V., Malivet C., Chacun M., Jan F., Tillequin S., Michel M., Koch B. (2001). Marked antitumor activity of a new potent acronycine derivative in orthotopic models of human solid tumors. Clin. Cancer Res..

